# Medication-Related Osteonecrosis of the Jaw: A Systematic Review and a Bioinformatic Analysis

**DOI:** 10.3390/ijms242316745

**Published:** 2023-11-25

**Authors:** Galina Laputková, Ivan Talian, Vladimíra Schwartzová

**Affiliations:** 1 Department of Medical and Clinical Biophysics, Faculty of Medicine, University of P. J. Šafárik, Trieda SNP 1, 040 11 Košice, Slovakia; ivan.talian@upjs.sk; 2 Clinic of Stomatology and Maxillofacial Surgery, Faculty of Medicine, University of P. J. Šafárik and Louis Pasteur University Hospital, 041 90 Košice, Slovakia; vladimira.schwartzova@upjs.sk

**Keywords:** osteonecrosis, systematic review, computational biology

## Abstract

The objective was to evaluate the current evidence regarding the etiology of medication-related osteonecrosis of the jaw (MRONJ). This study systematically reviewed the literature by searching PubMed, Web of Science, and ProQuest databases for genes, proteins, and microRNAs associated with MRONJ from the earliest records through April 2023. Conference abstracts, letters, review articles, non-human studies, and non-English publications were excluded. Twelve studies meeting the inclusion criteria involving exposure of human oral mucosa, blood, serum, saliva, or adjacent bone or periodontium to anti-resorptive or anti-angiogenic agents were analyzed. The Cochrane Collaboration risk assessment tool was used to assess the quality of the studies. A total of 824 differentially expressed genes/proteins (DEGs) and 22 microRNAs were extracted for further bioinformatic analysis using Cytoscape, STRING, BiNGO, cytoHubba, MCODE, and ReactomeFI software packages and web-based platforms: DIANA mirPath, OmicsNet, and miRNet tools. The analysis yielded an interactome consisting of 17 hub genes and hsa-mir-16-1, hsa-mir-21, hsa-mir-23a, hsa-mir-145, hsa-mir-186, hsa-mir-221, and hsa-mir-424. A dominance of cytokine pathways was observed in both the cluster of hub DEGs and the interactome of hub genes with dysregulated miRNAs. In conclusion, a panel of genes, miRNAs, and related pathways were found, which is a step toward understanding the complexity of the disease.

## 1. Introduction

Medication-related osteonecrosis of the jaw (MRONJ) encompasses osteonecrosis of the jaw associated with bisphosphonates, denosumab, and anti-resorptive agents [1,2,3,4]. It is a rare but serious drug reaction associated often with receiving high doses of anti-angiogenic and anti-resorptive medication, including mTOR inhibitors [5,6,7]. Anti-resorptive drugs, namely bisphosphonate and denosumab, are monoclonal antibodies that target the receptor activator of the tumor necrosis factor ligand superfamily member 11 [8,9]. Under conditions of accelerated skeletal turnover, bisphosphonates are selectively absorbed at sites of active bone remodeling [10]. Anti-angiogenic drugs, namely sunitinib and bevacizumab, are humanized monoclonal antibodies that impede the creation of novel blood vessels by suppressing the function of tyrosine kinases and vascular endothelial growth factor (VEGFA) [11].

MRONJ is a recognized phenomenon in nearly 1% of cancer patients and in 0.1% of those suffering from metabolic bone diseases [12]. The prevalence of MRONJ reported in studies varies widely, with incidence rates ranging from as low as 0.01% following low-dose oral bisphosphonate therapy to as high as 14.4% in high dose intravenous bisphosphonate treatment [13]. Dental extractions, implant procedures, oral and maxillofacial surgeries, periodontal disease, and invasive periodontal procedures have been identified as risk factors for MRONJ, with local inflammation being of greatest importance [14]. The etiology of MRONJ is multifactorial, encompassing multiple deficiencies that synchronize to result in bone resorption suppression [15], infection/inflammation [16], immune system dysfunction [17], angiogenesis inhibition [18], soft tissue toxicity [19], and systemic disorders related to conditions such as rheumatoid arthritis or diabetes mellitus [20]. Although there is no conclusive pathophysiology supported by scientific data, numerous fundamental queries persist. Even though there has been a decades-long investigation, the exact reason MRONJ is more frequently observed in the jawbone remains unclear.

Oral disorders encompass a range of conditions with worldwide prevalence and clinical significance. These ailments can have mutilating effects and significantly diminish the quality of life, as they affect a restricted area with critical physiological and social functions. Although crucial, several oral illnesses remain inadequately understood and frequently receive ineffective treatment. It is crucial to have a comprehensive understanding of the mechanisms at play in oral diseases to identify dependable, mechanistic indicators of clinical results, establish targeted therapeutic strategies, and customize prevention and treatment techniques. Conventional analysis of diseases only provides surface-level interpretations. Gaining a comprehensive knowledge of complex human disorders necessitates collating all pertinent data and scrutinizing biomarkers that are genetically associated with disease susceptibility. To facilitate the identification of innovative underlying molecular disease mechanisms, unbiased screening methods have been employed at various molecular levels to produce large-scale datasets. Numerous biological research areas, such as the investigation of oral conditions, gain advantages from recognizing these processes and biomarkers in single-omics analysis [21]. The application of multi-omics approaches, including genomics, transcriptomics, proteomics, and metabolomics, provides comprehensive molecular insights beyond single-omics methods. Therefore, the analysis aimed to pool existing data on the pathophysiological processes of MRONJ in humans, provided by multi-omics techniques such as high-throughput sequencing, gene expression arrays, and mass spectroscopy, to identify groups of biomarkers differentially expressed between cohorts and worthy of further investigation. At the same time, the objective of the work was to reveal altered signaling pathways and to create a multidimensional, layered configuration of MRONJ that would provide new insights into its pathobiology.

## 2. Materials and Methods

The results presented in this systematic analysis followed the Preferred Reporting Items for Systematic Reviews and Meta-Analyses (PRISMA 2020) guidelines [22].

### 2.1. Study Selection

#### 2.1.1. Inclusion Criteria

To be included, studies had to involve human oral mucosa, blood, serum, saliva, or adjacent bone or periodontium exposed to anti-resorptive or anti-angiogenic agents. The date of publication was not restricted, but only English language articles were considered.

#### 2.1.2. Exclusion Criteria

Studies that have been retracted previously, along with reviews, conference abstracts, case reports, letters to the editor, case studies, and studies involving non-human animal studies, have been excluded.

#### 2.1.3. Screening Process

**Figure 1:**
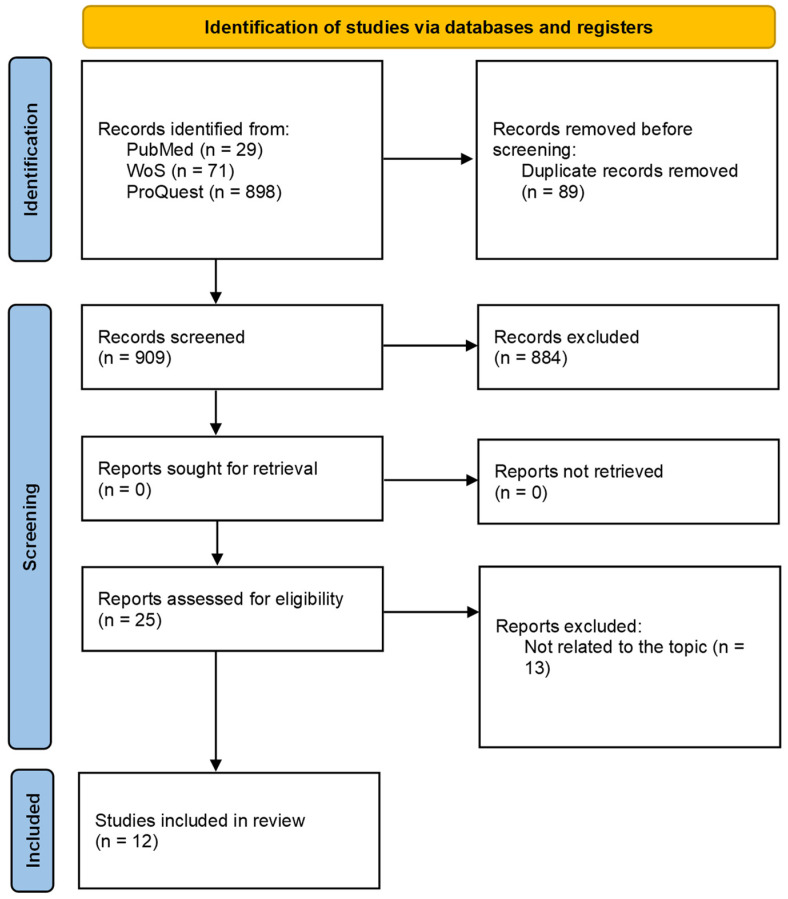
PRISMA flow diagram of literature for a systematic review on the screening of medication-induced osteonecrosis of the jaw.

The screening process was conducted with predetermined, objective inclusion criteria after the completion of a literature search. Figure 1 represents the procedure outlined. The survey was conducted between 7 and 10 March 2023. From inception until April 2023, two evaluators independently searched the databases of PubMed, Web of Science (WoS), and ProQuest. The data were pooled from those source articles that were related to mRNAs, microRNAs (miRNAs), proteins, and metabolites. The study merged key phrases with the logical operator “OR” and the results with the logical operator “AND”. The following terms were used in the search strategy: “mronj”, “bronj”, “aronj”, “dronj”, “medication-related osteonecrosis of the jaw”, “bisphosphonate-related osteonecrosis of the jaw”, “antiresorptive agent-related osteonecrosis of the jaw”, and “denosumab-related osteonecrosis of the jaw”. In addition, the search terms for transcriptomics were “microRNA” or “miRNA” and “transcriptome”, “transcriptomics” or “mRNA”. Proteomics was searched using the terms “proteomics” or “proteome”, while metabolomics was obtained by searching for “metabolome”, “metabolomics” or “metabolite”. No year restrictions were applied for article publication.

Further, the screening process involved manually removing duplicate results from the analysis. Two reviewers independently analyzed the titles and abstracts of the papers and evaluated the remaining articles to determine their eligibility. If a study’s suitability could not be determined solely from its title and abstract, its full text was examined. Citations for the included papers were tracked using Google Scholar or PubMed. A manual review of the reference lists of the included articles was conducted to select relevant articles. The results were then summarized from the articles that satisfied the inclusion criteria. The Revised Cochrane risk-of-bias tool for randomized trials (RoB 2, https://www.riskofbias.info/welcome/rob-2-0-tool/current-version-of-rob-2, accessed on 14 November 2023) was used to assess the risk of bias in domains related to the randomization process, deviations from the intended interventions, missing outcome data, measurement of the outcome, and selection of the reported result.

### 2.2. Data Analysis

#### 2.2.1. Gene Ontology Enrichment Analysis

The GeneCards human gene database (https://www.genecards.org, accessed on 10 July 2023) was used to verify and revise the list of differentially expressed genes (DEGs) compiled from eligible articles. Protein accession numbers were mapped to genes using the UniProt mapping tool (https://www.uniprot.org, accessed on 14 July 2023) [23]. The downloaded matching genes were then used for further investigation.

To investigate DEGs for overrepresentation in the hierarchical gene ontology (GO), the extensions of Cytoscape 3.10.0 [24] and BiNGO 3.0.5 [25] were used. The enrichment analysis for cell components, molecular function, and biological process terms was performed using the Benjamini and Hochberg multiple testing procedure. The significance level was set at 0.05 (*p* < 0.05).

#### 2.2.2. Protein–Protein Interaction Network and Module Analysis

The network of DEGs was created using the Search Tool for the Retrieval of Interacting Genes (STRING) database, which presents both insights and predictions regarding protein–protein interactions (PPIs). The network itself was constructed with the help of StringApp, Version 2.0.1 [26].

CytoHubba 0.1 [27] and Molecular Complex Detection (MCODE) 2.0.3 [28] allowed the exploration of hub genes and clusters within the network. All CytoHubba plug-in algorithms, including Maximum Neighborhood Component (MNC), Maximal Clique Centrality (MCC), Density of Maximum Neighborhood Component (DMNC), Degree (Deg), Betweenness (BC), Bottleneck (BN), Closeness (Clo), EcCentricity (EC), Edge Percolated Component (EPC), Stress (Str), and Radiality (Rad), were used to detect the hub genes [27]. The MCODE clustering was performed with a degree cutoff of 2, a node score cutoff of 0.2, a maximum depth of 20, and a k-score of 5.

#### 2.2.3. Pathway Enrichment Analysis

The ReactomeFI plug-in pathway database version 8.0.6 [29] was used for pathway enrichment analysis.

The mirPath v.4 database from DIANA Tools (https://diana-lab.e-ce.uth.gr/app/miRPathv4, accessed on 17 July 2023) was utilized to identify genes that could serve as miRNA target candidates.

#### 2.2.4. Multi-Omics Network

The multi-omics data were integrated using web-based platforms such as OmicsNet (https://www.omicsnet.ca, accessed on 19 July 2023) [30] and miRNet (https://www.mirnet.ca, accessed on 21 July 2023) [31]. If the multi-omics network consisted of more than 3000 nodes, we implemented the minimum network setting, i.e., the algorithms that generate the minimum network connecting all specified nodes.

## 3. Results

### 3.1. Systematic Review of Screening for MRONJ

**Figure 2:**
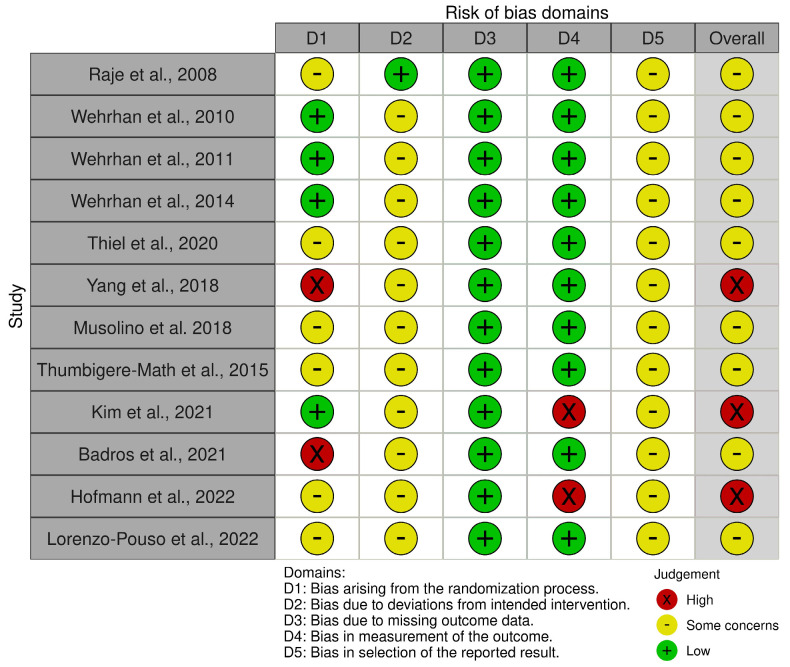
Risk of bias assessment [32,33,34,35,36,37,38,39,40,41,42,43]. According to the Revised Cochrane risk-of-bias tool for randomized trials (RoB 2, https://www.riskofbias.info/welcome/rob-2-0-tool/current-version-of-rob-2, accessed on 14 November 2023).

The search strategy produced 998 articles. Twenty-five articles underwent full-text review following a screening of their titles and abstracts. Twelve articles were ultimately included in the library after thirteen articles were excluded following a thorough examination of their full text (Figure 2). Exclusion criteria comprised such items as conference abstracts, letters, and review articles; non-human studies; and publications in languages other than English. Table 1 summarizes the characteristics of the studies on MRONJ that were included. Nine of the twelve studies that met the inclusion criteria were found to have an overall risk of bias of some concern, and three were found to have a high overall risk of bias.

No relevant studies have been found regarding the metabolomics of MRONJ.

### 3.2. Network Analysis of Protein Interaction Data

**Figure 3:**
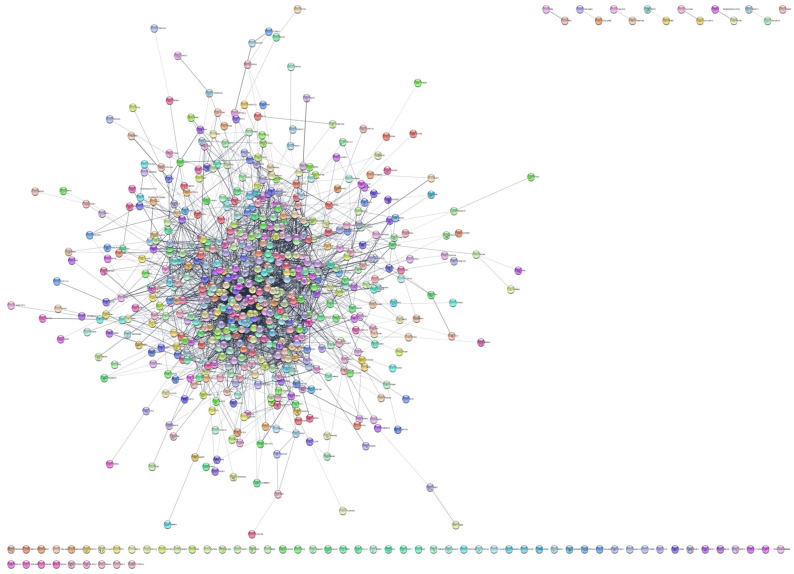
The STRING network formed from the dysregulated genes/proteins in MRONJ.

To investigate the protein interactions involved in MRONJ pathogenesis, we utilized STRING databases to analyze the 824 identified genes/proteins and created an interactive network via Cytoscape. Figure 3 depicts the resulting network consisting of 701 genes and 3993 edges, while Table 2 summarizes the network topology.

The network topology was analyzed using the cytoHubba (Version 0.1) extension of Cytoscape. The highly linked hub genes were extracted from the main complex network of DEGs by using the algorithms of cytoHubba. Subsequently, 24 genes were extracted that occurred at the intersection of at least three methods: *ALB*, *ANXA5*, *ATM*, *CCL2*, *CD44*, *CXCL8*, *CXCR4*, *EEF2*, *EGF*, *GART*, *HSP90AB1*, *HSPA4*, *IGF1*, *IL1B*, *IL6*, *ITGB1*, *JUN*, *LMNA*, *MMP9*, *PTPRC*, *RAB5A*, *RHOA*, *TNF*, and *VEGFA* (see Table 3).

In complement to the cytoHubba algorithms, MCODE clustering was employed to detect the molecular complexes and the seeds—the hub genes with a high degree of connectivity. In a complex PPI network of DEGs, MCODE identified five of the intra-connected regions/clusters and five hub genes/seeds with a high degree of connectivity (see Table 4).

In conjunction with cluster 1, the highest scoring MCODE clustering module, with the cytoHubba analysis results, a total of 17 hub genes were retrieved, comprising *ALB*, *CD44*, *CXCL2*, *CXCL8*, *CXCR4*, *EEF2*, *EGF*, *IGF1*, *IL1B*, *IL6*, *ITGB1*, *JUN*, *MMP9*, *PTPRC*, *RHOA*, *TNF*, and *VEGFA*.

### 3.3. GO Enrichment Analysis

A survey of how genes and gene products are represented in the biological domains concerning three aspects of molecular biology was conducted using Gene Ontology (GO). To associate GO terms with gene and protein sets, a series of enrichment analyses were performed in Cytoscape with the help of the BiNGO extension.

The dysregulated genes within the complex gene panel were linked to 422 GO terms, consisting of 333 biological process terms, 23 molecular function terms, and 66 cell component terms, as identified by GO enrichment analysis. The top GO enrichment terms associated with DEGs by *p*-values are shown in Table 5. The key molecular biological processes identified among these genes are those involved in regulating the immune system and the organism’s immune response. Numerous genes have been implicated in protein binding. DEGs were predominantly linked to the extracellular region and the extracellular space. 

A total of 918 GO terms were obtained from the analysis of seventeen hub genes. Of these GO terms, 893 were related to biological processes, 22 were associated with molecular function, and 23 were linked to cell component terms. Protein phosphorylation of amino acids constitutes a central molecular biological process. While protein binding was a common association among all hub genes, the top-ranked molecular function was cytokine receptor binding. DEGs were predominantly located in the extracellular space and region, as indicated in Table 6.

### 3.4. Multiomics Networks in MRONJ

**Figure 4:**
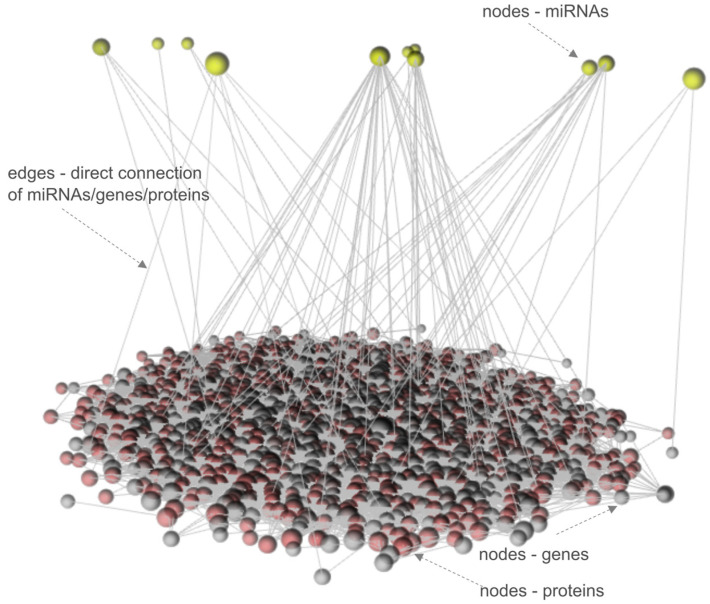
The multi-omics 3D layered network of MRONJ.

To investigate the fundamental mechanisms of MRONJ regulation, OmicsNet tools to visualize multi-layered networks with a 3D-based layered layout were used. We detected a complex intrinsic network that was eventually reduced to a minimally connected network consisting of 1300 nodes (1289 genes/proteins and 11 miRNAs) and 7816 edges after merging the initial set of 22 miRNAs, 550 genes, and 292 proteins (see Figure 4).

**Figure 5:**
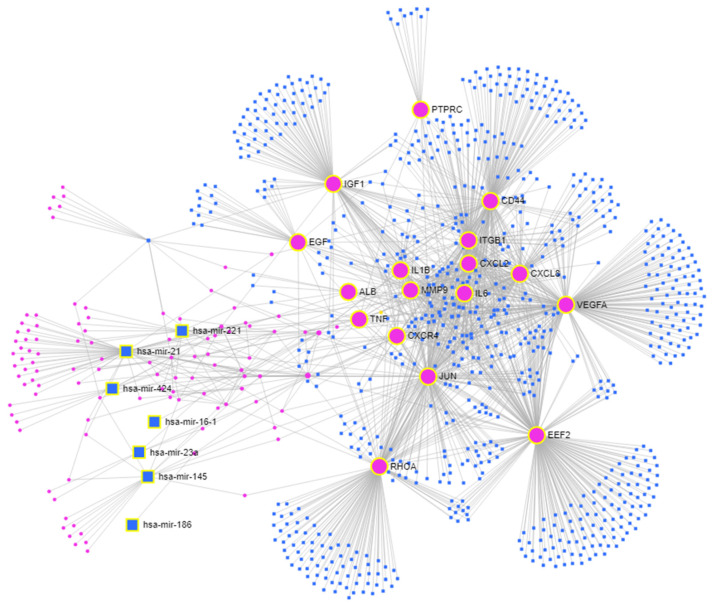
The multi-omics network of MRONJ. Input genes—large pink circles; input miRNAs—large blue squares; connector genes—small pink circles; connector miRNAs—small blue squares.

Next, we integrated the 17 genes shared between the MCODE cluster and cytoHubba analysis with the 22 miRNAs that were differentially expressed, ultimately producing a highly interconnected new network. This network produced an interactome of seventeen input genes, including *ALB*, *CD44*, *CXCL2*, *CXCL8*, *CXCR4*, *EEF2*, *EGF*, *IGF1*, *IL1B*, *IL6*, *ITGB1*, *JUN*, *MMP9*, *PTPRC*, *RHOA*, *TNF*, and *VEGFA*, and seven input miRNAs (hsa-mir-16-1, hsa-mir-21, hsa-mir-23a, hsa-mir-145, hsa-mir-186, hsa-mir-221, and hsa-mir-424) with connector genes/miRNAs using 1693 edges (Figure 5).

### 3.5. Pathway Enrichment Analysis

**Figure 6:**
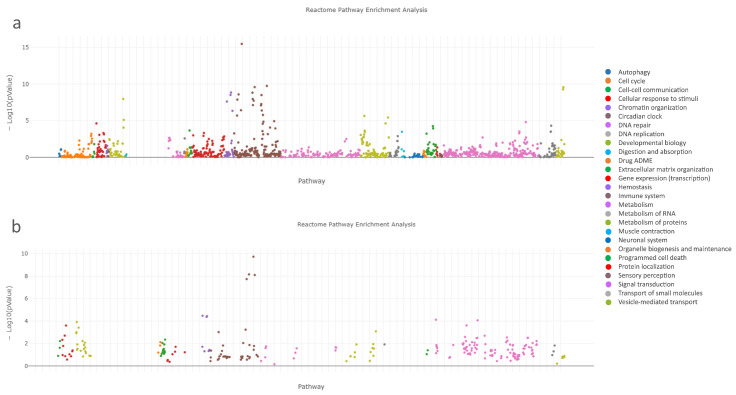
Pathway enrichment analysis of significantly dysregulated Reactome signaling pathways ranked according to the *p*-values; (**a**) the full set of dysregulated genes/proteins, (**b**) the set of hub genes/proteins of MRONJ.

To identify the pathways involved in medication-induced osteonecrosis of the jaw, we analyzed the associated genes/proteins of the complex panel of DEGs using the ReactomeFI tool within Cytoscape. Table 7 and Figure 6a indicate that DEGs were notably enriched in the top ten signaling pathways, specifically in the Innate immunity system pathways. These pathways form the nonspecific part of immunity and include functions such as Neutrophil degranulation (R-HSA-6798695) and regulation of the complement cascade (R-HSA-6803157). Similarly, pathway enrichment analysis was performed on the significantly dysregulated Reactome signaling pathways using the set of 17 hub genes/proteins identified in MRONJ (Table 8, Figure 6b).

To map the signaling pathways of MRONJ and to identify potential Reactome molecular pathway targets of miRNAs, the associated miRNAs (hsa-mir-16-1, hsa-mir-21, hsa-mir-23a, hsa-mir-145, hsa-mir-186, hsa-mir-221, and hsa-mir-424), obtained with the OmicsNet tools (Figure 5), underwent an in silico analysis with the DIANA Tools mirPath v.4 database. MiRNA-centric analysis of hsa-mir-16-1, hsa-mir-21, hsa-mir-23a, hsa-mir-145, hsa-mir-186, hsa-mir-221, and hsa-mir-424 was carried out with the help of the TarBase v8.0 database and miTarBase2022 as a secondary target source, searching for the direct miRNA target genes. The union of Reactome pathways option was used in the analysis. The results of the analysis are shown in Table 9. The Reactome pathways Interleukin-4 and interleukin-13 signaling (R-HSA-6785807) and signaling by interleukins (R-HSA-449147) show the most comprehensive association of miRNAs with gene targets consisting of *IL1B*, *VEGFA*, and *CXCL8*.

## 4. Discussion

To explore the pathological mechanisms of MRONJ, we utilized gene profiling datasets, proteins, and miRNAs. A variety of analytical strategies were employed to investigate the molecular mechanisms underlying MRONJ, including PPI network analysis, GO enrichment, and Reactome pathway enrichment analysis. 

Antiresorptive therapy, including bisphosphonates, denosumab, and angiogenesis inhibitors, may trigger MRONJ, which can affect both the maxilla and mandible [44,45]. Several hypotheses have been developed regarding the pathophysiology of MRONJ: (1) suppression of bone resorption; (2) inflammation and oral microbial infection; (3) inhibition of angiogenesis and anti-lymphangiogenesis; (4) dysfunction of innate or acquired immunity (T and B cells, macrophages, DCs, and natural killer cells); and (5) soft tissue toxicity are all potential adverse effects [46,47].

Pathway enrichment analysis is a valuable tool for gaining a mechanistic comprehension of the intricate gene, miRNA, and protein inventories resulting from omics experiments. It assists in the interpretation of biomedical data to reveal the molecular basis of disease [48]. Our analysis identified immune dysfunction-related pathways associated with MRONJ as the main reason for developing and progressing osteonecrosis. When analyzing the entire pool of genes and proteins, it is evident that the Reactome signaling pathways that are significantly dysregulated are primarily dominated by the Innate immune system and Neutrophil degranulation pathways, as indicated by the *p*-values. Reducing the set of hub genes to 17, which includes *ALB*, *CD44*, *CXCL2*, *CXCL8*, *CXCR4*, *EEF2*, *EGF*, *IGF1*, *IL1B*, *IL6*, *ITGB1*, *JUN*, *MMP9*, *PTPRC*, *RHOA*, *TNF*, and *VEGFA*, resulted in the dominance of cytokine signaling pathways in the immune system. Moreover, simultaneously analyzing hub genes with miRNAs (hsa-mir-16-1, hsa-mir-21, hsa-mir-23a, hsa-mir-145, hsa-mir-186, hsa-mir-221, and hsa-mir-424) using network-based multiomics analysis revealed dysregulated pathways in the immune system’s cytokine signaling, specifically signaling by interleukins. 

There is increasing evidence that inflammatory osteoimmunology is critical to the development of osteonecrosis [49]. Cytokines that regulate inflammatory responses contribute to the onset and progression of osteonecrosis [17,50,51]. In individuals with osteonecrosis, necrotic cells produce cytokines that attract inflammatory cells, triggering both local and systemic immune responses [17,52,53].

The function of immune cells and bone-forming cells, especially osteoblasts and osteoclasts, is regulated by cytokines, inflammatory chemokines, and growth factors [54]. Research confirms the involvement of cytokine networks in osteoclast differentiation and regulation. Cytokines, such as tumor necrosis factor-alpha, interleukins 1, -6, -7, -8, -11, -15, -17, -23, and -34, facilitate the process of osteoclast differentiation. By contrast, anti-osteoclastogenic cytokines, namely interferons alpha, beta, and gamma and interleukins 3, -4, -10, -12, -27, and -33, suppress osteoclasts. [55].

The pathogenesis of inflammatory bone disease is significantly influenced by T cells and B cells [56]. There have been intense discussions regarding new roles for B cells and a potential role for peripheral blood γδ T cells [57,58]. Γδ T cells are innate lymphocytes with a crucial role in regulating immune homeostasis [59]. Kalyan et al. investigated the potential predictive role of peripheral blood γδ T cells in osteonecrosis of the jaw. The authors propose that the loss of γδ T cells caused by bisphosphonates may be involved in the development of osteonecrosis [57]. Moreover, the proliferation of macrophages and γδ T cells promotes inflammation in zoledronic acid-induced jaw necrosis, as the authors of the study [60] concluded. 

The understanding of biological systems is facilitated by the objective study of PPIs. An effective approach to characterizing system-wide PPIs is the use of PPI networks. These networks are constructed from pairwise protein interactions and serve as an efficient tool for describing PPI landscapes [61]. To investigate protein functions and biological processes based on predicted PPIs and to gain new insights into diseases, the DEG PPI network was analyzed in this study. For osteonecrosis of the jaw, 17 hub genes with aberrant expression were selected. They included *ALB*, *CD44*, *CXCL2*, *CXCL8*, *CXCR4*, *EEF2*, *EGF*, *IGF1*, *IL1B*, *IL6*, *ITGB1*, *JUN*, *MMP9*, *PTPRC*, *RHOA*, *TNF*, and *VEGFA*.

Various signaling molecules, such as *VEGFA*, *EGF*, *MMP9*, and *TNF*, contribute to angiogenesis by stimulating the proliferation and migration of vascular endothelial cells [62]. *VEGFA* is a highly potent pro-angiogenic factor that plays a critical role in the healing of microvascular wounds associated with bisphosphonate administration [63]. A statistically significant increase in *VEGFA* gene expression was also demonstrated in response to zoledronic acid [64]. The physiological processes of angiogenesis and vascular remodeling involve the regulation of non-coding RNAs, specifically miRNA-based regulation (as noted by reference [65]). Objective evaluation of these processes is necessary for accurate understanding.

MicroRNAs are small endogenous RNA molecules (∼22 nt) that were recently discovered. Disorders such as cancer or heart disease have demonstrated the diagnostic potential of circulating miRNAs [66,67]. MiRNA-mediated RNA interference, a unique mechanism that binds miRNAs to different direct targets, controls both post-transcriptional gene expression and protein expression [68].

Our OmicsNet network analysis generated an interactome of input genes including *ALB*, *CD44*, *CXCL2*, *CXCL8*, *CXCR4*, *EEF2*, *EGF*, *IGF1*, *IL1B*, *IL6*, *ITGB1*, *JUN*, *MMP9*, *PTPRC*, *RHOA*, *TNF*, and *VEGFA* with input miRNAs comprising hsa-mir-16-1, hsa-mir-21, hsa-mir-23a, hsa-mir-145, hsa-mir-186, hsa-mir-221, and hsa-mir-424. Further analysis focused on miRNA identified the most extensively linked miRNAs targeting *IL1B*, *VEGFA*, *CXCL8*, and *CD44* directly (Table 9).

MiR-145 targets mainly the interleukins [38] and is also implicated in M2 macrophage polarization [69]. Silencing miR-145 leads to the advancement of femoral head regeneration by upregulating *VEGFA* [70].

The PPI network revealed that *CXCL8* [71] is among the factors related to the progression of osteonecrosis of the jaw, and our analysis identified it as a direct target of miR-23a. Earlier studies reported a significant reduction of miR-23a-3p during osteogenic differentiation of human mesenchymal stem cells of bone marrow origin [72]. Furthermore, inhibition of miR-23a in a rat model resulted in a lower incidence of osteonecrosis [73].

Serum microRNAs, including miR-21, miR-23a, and miR-145, were observed to be dysregulated in BRONJ rats [37]. Furthermore, candidate microRNA expressions were confirmed in human samples. During the progression of BRONJ, there was an upregulation of circulating miR-21, which corresponds to the alteration of miR-21 in pro-osteoclastogenesis [37].

## 5. Conclusions

The emergence of high-throughput platforms for the comprehensive analysis of genes, proteins, and other biological molecules has afforded an exceptional capability for the recognition of novel, valid signatures of disease-related processes.

In conclusion, our systematic review study indicates specific alterations in proteins, genes, and microRNAs and thus unravels novel insights into the molecular mechanism behind the MRONJ disease. The identified dysregulated genes in MRONJ are mostly linked to the regulation of immune system processes and the immune response of the organism. These dysregulated genes significantly enrich pathways related to the Innate immunity system, a crucial component of the nonspecific part of immunity. Particularly important are the 17 hub genes, which exert dominance in the cytokine signaling pathways within the immune system. Additionally, the interaction network between these hub genes and dysregulated miRNAs uncovered pathways associated with the cytokine signaling in the immune system, particularly the signaling by interleukins pathway. Subsequent miRNA analysis showed a set of highly connected miRNAs with direct targeting of multiple genes such as *IL1B*, *VEGFA*, *CXCL8*, and *CD44*.

This study has potential limitations that should be noted. There was considerable heterogeneity observed between studies, which may impact the interpretation of the results. Factors such as patient selection variability, differences in the origins and causes of MRONJ, and variations in the material and methodology used could contribute to this heterogeneity. To ensure more reliable results, it would be beneficial to establish strict inclusion/exclusion criteria based on the disease state and MRONJ treatment in future studies. Additionally, our objective was to present a comprehensive overview of the pathophysiological processes of MRONJ in humans using multi-omics techniques. Expanding the research to encompass other types of non-coding RNAs could prove helpful in filling this gap. Furthermore, there is a shortage of data for analysis due to the limited amount of metabolomics research.

Despite the limitations of this study, the panel of proteins, genes, and microRNAs presented, along with their associated pathways, constitutes a significant advancement toward comprehending the intricate cause of MRONJ.

## Figures and Tables

**Table 1:** Characteristics of included studies on medication-induced osteonecrosis of the jaws.

mRNA		
Reference	Sample Type	Method
Raje et al., 2008, 10.1158/1078-0432.CCR-07-1430 [32]	Peripheral blood mononuclear cells. Patients: MM patients with ONJ (*n* = 8). Controls: MM patients without ONJ (*n* = 10), healthy volunteers (*n* = 5).	Affymetrix U133Plus 2.0 Gene Chip (Affymetrix, Santa Clara, CA, USA).
Wehrhan et al., 2010, 10.1186/1479-5876-8-96 [33]	Periodontal samples. Patients: patients with BRONJ (*n* = 20). Controls: non-BP exposed periodontal samples (*n* = 20).	Microfluid Lab-on-a-Chip technology (Agilent RNA 6000 Pico Kit and the Agilent 2100 Bioanalyzer, Agilent, Waldbronn, Germany). The cDNAs from total RNA were synthesized using the High-Capacity cDNA Archive Kit (Cat. 4322171; Applied Biosystems, Foster City, CA, USA). Real-time RT qPCR (QuantiTect Primer Assay; Qiagen, Hilden, Germany).
Wehrhan et al., 2011, 10.1111/j.1601-0825.2010.01778.x [34]	Periodontal samples. Patients: patients with BRONJ (*n* = 20). Controls: non-BP exposed periodontal samples (*n* = 20).	Microfluid Lab-on-a-Chip technology (Agilent RNA 6000 Pico Kit and the Agilent 2100 Bioanalyzer, Agilent, Waldbronn, Germany). The cDNAs from total RNA were synthesized using the High-Capacity cDNA Archive Kit (Cat. 4322171; Applied Biosystems, Foster City, CA, USA). Real-time RT qPCR (QuantiTect Primer Assay; Qiagen, Hilden, Germany).
Wehrhan et al., 2014 10.1007/s00784-014-1354-7 [35]	Jawbone samples. Patients: patients with BRONJ (*n* = 15). Controls: non-BP exposed samples (*n* = 20).	Total RNA extraction (RNeasy Kit, Qiagen, Hilden, Germany). Microfluid Lab-on-a-Chip technology (Agilent RNA 6000 Pico Kit and the Agilent 2100 Bioanalyzer, Agilent, Waldbronn, Germany). High-capacity cDNA Archive Kit (Cat. No. 4322171; Applied Biosystem, Foster City, CA, USA). Real-time RT quantitative PCR analyses: Hs_SPP1_1_SGQuantiTect Primer Assay (200) on the ABI Prism 7300 Sequence Detection System (Applied Biosystems, Waltham, MA, USA). PCR amplification: the QuantiTect TM SYBR^®^ green PCR kit (Cat. No. 204143; Qiagen, Hilden, Germany).
Thiel et al., 2020 10.1016/j.prp.2020.153245 [36]	Jawbone samples. Patients: diagnosed with MRONJ (*n* = 12). Controls: subjects without MRONJ (*n* = 6).	RNA extraction kit (miRNeasy Mini Kit; Qiagen, Hilden, Germany). The total RNA was reverse transcribed into cDNA using the iScript™ cDNA Synthesis Kit (Bio-Rad, Hercules, CA, USA). PCR amplification: SsoAdvanced™ Universal SYBR^®^ Green Supermix (Bio-Rad, Hercules, CA, USA) Amplification was conducted on the CFX Connect Real-Time PCR System (Bio-Rad, Hercules, CA, USA).
**miRNA**		
**Reference**	**Sample Type**	**Method**
Raje et al., 2008, 10.1158/1078-0432.CCR-07-1430. [32]	Peripheral blood mononuclear cells. Patients: MM patients with ONJ (*n* = 8). Controls: MM patients without ONJ (*n* = 10), healthy volunteers (*n* = 5).	Affymetrix U133Plus 2.0 Gene Chip (Affymetrix, Santa Clara, California, USA).
Yang et al., 2018, 10.7150/ijms.27593 [37]	Serum. Patients: patients with BRONJ (*n* = 6). Controls: non-BP healthy individuals (*n* = 11).	RNA extraction: mirVana Paris Kit (Ambion, Huntingdon, Cambridgeshire, United Kingdom). The microRNAs were reversed to cDNA using the miScript II RT Kit (Qiagen, Hilden, Germany). Q-RT-PCR analysis was conducted using the miScript SYBR Green PCR Kit (Qiagen, Hilden, Germany) with a 7500 Real-Time PCR System (Applied Biosystems, Foster City, CA, USA).
Musolino et al. 2018, 10.1007/s00277-018-3296-7 [38]	Peripheral blood. Patients: MM patients with ONJ (*n* = 5). Controls: healthy volunteers (*n* = 5).	RNA extraction: the Total Purification Plus Kit (Norgen Biotek Corporation, Thorold, ON, Canada). Total RNA was transcribed into cDNA through an All-in-One miRNA first-strand cDNA synthesis kit (GeneCopoeia Inc., Rockville, MD, USA). Real-Time qPCR employed a 7500 Real-Time PCR System (Applied Biosystems, Foster City, CA, USA).
**Proteins**		
**Reference**	**Sample Type**	**Method**
Thumbigere-Math et al., 2015, 10.1111/odi.12204 [39]	Saliva. Patients: BRONJ (*n* = 20), high- and low-infusion groups. Controls: non-BRONJ patients (*n* = 20).	iTRAQ labeling was followed by fractionation using strong cation exchange chromatography, and fractions were analyzed by reversed-phase microcapillary LC-S (LTQ-Orbitrap).
Kim et al., 2021, 10.7150/ijms.61552 [40]	MG-63, SCC-9, SCC-15, and HUVEC cells. ALN-treated and non-ALN control groups.	2D-DIGE, followed by MALDI TOF/TOF MS (4800 Plus, Applied Biosystems, Foster City, CA, Life Sciences, USA).
Badros et al., 2021, 10.3389/fonc.2021.704722 [41]	Saliva, serum. Patients: MM patients who underwent intravenous BP therapy and developed BRONJ (*n* = 14). Controls: non-BRONJ MM patients (*n* = 96).	Luminex™ technology (EMD Millipore, Burlington, MA, USA).
Hofmann et al., 2022, 10.1007/s10266-022-00691-y [42]	HAOB cells. BEV/SUN-treated and non-BEV/SUN control groups.	ELISA
Lorenzo-Pouso et al., 2022, 10.1111/odi.14201 [43]	Saliva. Patients: Group 1—MRONJ cases (*n* = 18). Controls: Group 2—individuals undergoing treatment with BMAs for more than 24 months without MRONJ (*n* = 10). Group 3—healthy volunteers (*n* = 10).	SDS-PAGE, shotgun DDA by micro-flow LC-MS/MS, a quadrupole-TOF mass spectrometer (Triple TOF 6600 [SCIEX, Framingham, MA, USA]) working in ESI + performed DDA analysis.

ALN—alendronate, BEV—bevacizumab, BP—bisphosphonate; BMAs—bone-modifying agents, BRONJ—bisphosphonate-related osteonecrosis of the jaw, DDA—data-dependent acquisition, DEP—differentially expressed proteins, ELISA—enzyme-linked immunoabsorbance assay, iTRAQ—isobaric tags for relative and absolute quantitation, LC—liquid chromatography, MM—multiple myeloma, MS—mass spectrometry, ONJ—osteonecrosis of the jaw, SDS-PAGE—sodium dodecyl-sulfate polyacrylamide gel electrophoresis, SUN—sunitinib, TOF—time-of-flight.

**Table 2:** The most relevant topological parameters of the STRING network.

Topological Parameters	Values
The average number of neighbors	12.755
Clustering coefficient	0.264
Characteristic path length	3.294
Network diameter	9
Number of edges	3993
Number of nodes	701

**Table 3:** Hub genes identified by the cytoHubba plug-in algorithms. The crucial hub genes that occurred at the intersection of at least three methods are highlighted in red.

MCC	DMN	MNC	Degree	FPC	Bottleneck	EcCentricity	Closeness	Radiality	Betweenness	Stress	Clustering Coefficient
*ALB*	*A1BG*	*ALB*	*ALB*	*ALB*	*ALB*	*ARF1*	*ALB*	*ALB*	*ALB*	*ALB*	*A2ML1*
*ANXA5*	*AGER*	*ANXA5*	*ANXA5*	*ANXA5*	*CAT*	*ARHGDIA*	*ANXA5*	*ANXA5*	*ATM*	*ATM*	*BANK1*
*CCL2*	*ANGPT1*	*B2M*	*CCL2*	*CCL2*	*CD44*	*ARRB1*	*ATM*	*ATM*	*CD44*	*CD44*	*CFHR5*
*CD44*	*CD83*	*CCL2*	*CD44*	*CD44*	*CXCL8*	*BCL2L11*	*CCL2*	*CASP8*	*CLTC*	*CXCL8*	*DPT*
*CSF3*	*CXCL1*	*CD44*	*CXCL8*	*CXCL8*	*EEF1A1*	*BTK*	*CD44*	*CD44*	*EEF2*	*EEF2*	*ENSP00000330898*
*CXCL8*	*CXCL2*	*CXCL8*	*CXCR4*	*CXCR4*	*EGF*	*CD83*	*CXCL8*	*CXCL8*	*EGF*	*EGF*	*ENSP00000377747*
*CXCR4*	*EEF1B2*	*CXCR4*	*EEF2*	*EGF*	*FUS*	*COL1A1*	*CXCR4*	*CXCR4*	*GART*	*GART*	*FAM213A*
*EGF*	*EIF2S3*	*EGF*	*EGF*	*HSPA4*	*GART*	*CYCS*	*EGF*	*CYCS*	*HIST1H4F*	*HSP90AB1*	*GTF3C4*
*IGF1*	*EIF5A2*	*HSP90AB1*	*HSP90AB1*	*IGF1*	*HIST1H4F*	*DNAJB1*	*HSP90AB1*	*EGF*	*HSP90AB1*	*HSPA4*	*CHI3L1*
*IL1B*	*CHI3L1*	*HSPA4*	*HSPA4*	*IL1B*	*HSP90AB1*	*FAS*	*HSPA4*	*HSP90AB1*	*HSPA4*	*IL1B*	*IL36A*
*IL6*	*LRG1*	*IGF1*	*IGF1*	*IL6*	*HSPA4*	*FCGR3A*	*IGF1*	*HSPA4*	*IL6*	*IL6*	*KRT76*
*JUN*	*MMP1*	*IL1B*	*IL1B*	*ITGB1*	*IGF1*	*GART*	*IL1B*	*IGF1*	*JUN*	*JUN*	*LMF1*
*KDR*	*ORM1*	*IL6*	*IL6*	*JUN*	*JUN*	*IL6*	*IL6*	*IL1B*	*LMNA*	*LMNA*	*ME1*
*MMP9*	*ORM2*	*ITGB1*	*ITGB1*	*MMP9*	*KRT14*	*ITGB1*	*ITGB1*	*IL6*	*PTPRC*	*PTPRC*	*NOV*
*PTGS2*	*PSMC1*	*JUN*	*JUN*	*PTGS2*	*LMNA*	*KRT19*	*JUN*	*JUN*	*RAB5A*	*RAB5A*	*POLR2J3*
*PTPRC*	*RPLP1*	*MMP9*	*MMP9*	*PTPRC*	*PTPRC*	*NR4A2*	*MMP9*	*MMP9*	*RHOA*	*RHOA*	*SEL1L3*
*SPP1*	*RPLP2*	*PTPRC*	*PTPRC*	*RHOA*	*RAB5A*	*PPP2CB*	*PTPRC*	*PTPRC*	*SRSF1*	*TFRC*	*SELM*
*TGFB1*	*SAA4*	*RHOA*	*RHOA*	*SPP1*	*RHOA*	*SAA4*	*RHOA*	*RHOA*	*TFRC*	*TNF*	*SERPIND1*
*TNF*	*SERPIND1*	*TNF*	*TNF*	*TNF*	*SRSF1*	*TXN*	*TNF*	*TNF*	*TNF*	*VEGFA*	*TNN*
*VEGFA*	*TNFRSF11B*	*VEGFA*	*VEGFA*	*VEGFA*	*TNF*	*VEGFA*	*VEGFA*	*VEGFA*	*VEGFA*	*YWHAZ*	*VPS36*

**Table 4:** MCODE-interconnected clusters generated from the Cytoscape-derived gene interaction network. The seed node with the highest score within the cluster is marked with an asterisk.

Cluster	Score (Density * Nodes)	Nodes	Edges	Node IDs
1	24.794	64	781	BGLAP, BMP2, CAT, CCL4, CCT2, CD44, COL1A1, CSF3, CXCL1, CXCL2, CXCL8, CXCR4, CYCS, EEF1A1, EEF1B2, EEF1D, EEF1G, EEF2, EGF, EIF2S3, EIF5A, EIF5A2, FGG, IGF1, IL1B, IL6, ITGB1, JUN, KDR, MARS, MMP1, MMP8, MMP9, NT5E, PSMC1, PTGS2, PTPRC, RHOA, RPL10, RPL12, RPL27A, RPL4, RPLP1, RPLP2, RPS10, RPS12, RPS16, RPS23, RPS25, RPSA, RUNX2, SERPINA1, SERPINC1, SOD2, SPP1, TGFB1, TNF *, TNFRSF11B, TNFSF11, TPT1, VEGFA
2	13.429	43	282	A1BG *, A2M, AGER, AMBP, ANXA5, APOA2, APOB, APOH, ATM, AZGP1, BCL2L11, C3, C4B, CASP8, CCL2, CP, CREB1, FAS, FCGR3A, FOXO1, GART, GIG25, HP, HPX, HSP90AB1, HSPA4, HSPB1, ITIH2, ITIH4, JAK1, KLRK1, LCK, LCN2, LRG1, MCL1, NFATC1, ORM1, ORM2, PDGFB, TF, TFRC, TTR, TXN
3	10.133	16	76	DSG1, DSP, IVL, KRT14, KRT15, KRT16, KRT17, KRT4, KRT5, KRT6B, KRT6C, SCEL, SPRR1A *, SPRR1B, SPRR3, TGM1
4	6.933	31	104	ACTG2, ALAS2, ATRX, CA2, CBFB, DDX3X, ETS1, GATA2, H2AFJ, HBA1, HBA2, HBB *, HBD, HBG1, HBG2, HIST1H1B, HIST1H1E, HIST1H2AB, HIST1H2AC, HIST1H3J, KMT2A, MYL12A, MYL6, SLC25A37, SLC4A1, SRSF1, SUPT16H, TAL1, TPM2, TPM3, TPM4
5	6	6	16	CELF1, FUS, HNRNPK *, MBNL1, SRSF10, SRSF3

**Table 5:** Gene ontology enrichment analysis performed in Cytoscape using the BiNGO extension. The full set of dysregulated genes/proteins was considered in the analysis. The most enriched gene ontology terms based on the respective *p*-values are depicted.

GO-ID	Description	*p*-Value	Corr *p*-Value	x	n	X	N
Biological Process							
2376	immune system process	2.9469 × 10^−15^	1.0179 × 10^−11^	97	947	631	14,265
6950	response to stress	1.7284 × 10^−13^	2.9850 × 10^−10^	143	1771	631	14,265
9611	response to wounding	4.6100 × 10^−13^	5.3076 × 10^−10^	64	541	631	14,265
6955	immune response	2.5317 × 10^−12^	2.1861 × 10^−9^	68	618	631	14,265
6952	defense response	8.4706 × 10^−12^	5.1466 × 10^−9^	67	620	631	14,265
42221	response to a chemical stimulus	8.9402 × 10^−12^	5.1466 × 10^−9^	120	1462	631	14,265
48513	organ development	1.7962 × 10^−11^	8.8627 × 10^−9^	138	1792	631	14,265
48583	regulation of response to stimulus	3.1299 × 10^−11^	1.3514 × 10^−8^	59	524	631	14,265
9888	tissue development	1.3146 × 10^−10^	4.9607 × 10^−8^	73	750	631	14265
6954	inflammatory response	1.4362 × 10^−10^	4.9607 × 10^−8^	42	315	631	14,265
**Molecular Function**							
5515	protein binding	2.9969 × 10^−19^	2.6493 × 10^−16^	462	8106	667	15,404
5198	structural molecule activity	2.5854 × 10^−13^	1.1427 × 10^−10^	68	600	667	15,404
5488	binding	4.2458 × 10^−11^	1.2511 × 10^−8^	596	12,340	667	15,404
5200	structural constituent of the cytoskeleton	8.2347 × 10^−8^	1.8199 × 10^−5^	16	74	667	15,404
3823	antigen binding	1.5501 × 10^−7^	2.3636 × 10^−5^	14	59	667	15,404
4857	enzyme inhibitor activity	1.6043 × 10^−7^	2.3636 × 10^−5^	33	279	667	15,404
3746	translation elongation factor activity	9.3982 × 10^−7^	1.1869 × 10^−4^	8	20	667	15,404
4866	endopeptidase inhibitor activity	1.2753 × 10^−6^	1.4031 × 10^−4^	21	146	667	15,404
61135	endopeptidase regulator activity	1.4285 × 10^−6^	1.4031 × 10^−4^	21	147	667	15,404
30414	peptidase inhibitor activity	3.4059 × 10^−6^	3.0108 × 10^−4^	21	155	667	15,404
**Cell Component**							
5615	extracellular space	4.5890 × 10^−14^	2.1385 × 10^−11^	78	748	680	16,336
5576	extracellular region	1.9191 × 10^−13^	4.4715 × 10^−11^	151	2022	680	16,336
44421	extracellular region part	2.5524 × 10^−12^	3.9647 × 10^−10^	89	985	680	16,336
43228	non-membrane-bounded organelle	6.4518 × 10^−10^	6.0131 × 10^−8^	160	2425	680	16,336
43232	intracellular non-membrane-bounded organelle	6.4518 × 10^−10^	6.0131 × 10^−8^	160	2425	680	16,336
5737	cytoplasm	2.2730 × 10^−9^	1.7654 × 10^−7^	393	7634	680	16,336
5856	cytoskeleton	3.1182 × 10^−9^	2.0758 × 10^−7^	104	1399	680	16,336
1533	cornified envelope	1.0235 × 10^−8^	5.9620 × 10^−7^	10	23	680	16,336
31983	vesicle lumen	2.4577 × 10^−8^	1.2725 × 10^−6^	12	38	680	16,336
31093	platelet alpha granule lumen	1.0035 × 10^−7^	4.6761 × 10^−6^	11	35	680	16,336

**Table 6:** Gene ontology enrichment analysis in Cytoscape using the BiNGO extension. A set of seventeen hub-dysregulated genes/proteins was considered in the analysis. The most enriched gene ontology terms based on the respective *p*-values are depicted.

GO-ID	Description	*p*-Value	Corr *p*-Value	x	n	X	N
Biological Process							
1932	regulation of protein amino acid phosphorylation	5.3257 × 10^−11^	1.6700 × 10^−8^	8	217	17	14,306
42325	regulation of phosphorylation	5.5034 × 10^−11^	1.6700 × 10^−8^	10	518	17	14,306
42327	positive regulation of phosphorylation	8.2778 × 10^−11^	1.6700 × 10^−8^	7	131	17	14,306
19220	regulation of the phosphate metabolic process	8.5947 × 10^−11^	1.6700 × 10^−8^	10	542	17	14,306
51174	regulation of the phosphorus metabolic process	8.5947 × 10^−11^	1.6700 × 10^−8^	10	542	17	14,306
10562	positive regulation of the phosphorus metabolic process	9.7175 × 10^−11^	1.6700 × 10^−8^	7	134	17	14,306
45937	positive regulation of the phosphate metabolic process	9.7175 × 10^−11^	1.6700 × 10^−8^	7	134	17	14,306
35468	positive regulation of the signaling pathway	1.2071 × 10^−10^	1.8152 × 10^−8^	9	380	17	14,306
48661	positive regulation of smooth muscle cell proliferation	1.4481 × 10^−10^	1.9356 × 10^−8^	5	29	17	14,306
10647	positive regulation of cell communication	2.5305 × 10^−10^	3.0442 × 10^−8^	9	413	17	14,306
**Molecular Function**							
5126	cytokine receptor binding	3.1215 × 10^−8^	3.3196 × 10^−6^	6	186	17	15,443
5125	cytokine activity	4.3968 × 10^−8^	3.3196 × 10^−6^	6	197	17	15,443
8083	growth factor activity	6.2752 × 10^−7^	3.1585 × 10^−5^	5	160	17	15,443
70851	growth factor receptor binding	1.6672 × 10^−6^	6.2937 × 10^−5^	4	82	17	15,443
5102	receptor binding	2.3472 x× 10^−6^	7.0887 × 10^−5^	8	922	17	15,443
5515	protein binding	1.7881 × 10^−5^	4.4999 × 10^−4^	17	8122	17	15,443
17022	myosin binding	2.3660 × 10^−4^	5.1037 × 10^−3^	2	21	17	15,443
5518	collagen binding	7.8337 × 10^−4^	1.4786 × 10^−2^	2	38	17	15,443
8009	chemokine activity	1.1976 × 10^−3^	2.0093 × 10^−2^	2	47	17	15,443
42379	chemokine receptor binding	1.4643 × 10^−3^	2.1505 × 10^−2^	2	52	17	15,443
**Cell Component**							
5615	extracellular space	5.3421 × 10^−10^	4.8078 × 10^−8^	10	747	17	16,377
44421	extracellular region part	7.8189 × 10^−9^	3.5185 × 10^−7^	10	985	17	16,377
31093	platelet alpha granule lumen	4.0780 × 10^−8^	1.0316 × 10^−6^	4	35	17	16,377
60205	cytoplasmic membrane-bounded vesicle lumen	4.5849 × 10^−8^	1.0316 × 10^−6^	4	36	17	16,377
31983	vesicle lumen	5.7381 × 10^−8^	1.0329 × 10^−6^	4	38	17	16,377
31091	platelet alpha granule	1.9266 × 10^−7^	2.8900 × 10^−6^	4	51	17	16,377
9986	cell surface	7.8170 × 10^−7^	1.0050 × 10^−5^	6	340	17	16,377
30141	stored secretory granule	9.9195 × 10^−7^	1.1159 × 10^−5^	5	186	17	16,377
16023	cytoplasmic membrane-bounded vesicle	2.0006 × 10^−6^	2.0006 × 10^−5^	7	647	17	16,377
31988	membrane-bounded vesicle	2.4025 × 10^−6^	2.1623 × 10^−5^	7	665	17	16,377

**Table 7:** Pathway enrichment analysis of the Reactome signaling pathways dysregulated in MRONJ ranked according to their *p*-values. The full set of dysregulated genes/proteins was considered in the analysis.

Reactome Pathway ID	Name	FDR	*p*-Value	Number of Proteins in Pathway	Proteins from Gene Set
R-HSA-168249	Innate immune system	3.90 × 10^−13^	3.33 × 10^−16^	1155	120
R-HSA-6798695	Neutrophil degranulation	7.95 × 10^−8^	1.82 × 10^−10^	479	58
R-HSA-977606	Regulation of complement cascade	7.95 × 10^−8^	2.62 × 10^−10^	127	27
R-HSA-2168880	Scavenging of heme from plasma	7.95 × 10^−8^	2.72 × 10^−10^	92	23
R-HSA-2173782	Binding and uptake of ligands by scavenger receptors	1.25 × 10^−7^	5.35 × 10^−10^	122	26
R-HSA-114608	Platelet degranulation	2.54 × 10^−7^	1.43 × 10^−9^	128	26
R-HSA-166658	Complement cascade	2.54 × 10^−7^	1.52 × 10^−9^	138	27
R-HSA-5690714	CD22-mediated BCR regulation	3.74 × 10^−7^	2.64 × 10^−9^	70	19
R-HSA-76005	Response to elevated platelet cytosolic Ca2^+^	3.74 × 10^−7^	3.12 × 10^−9^	133	26
R-HSA-2029482	Regulation of actin dynamics for phagocytic cup formation	3.74 × 10^−7^	3.20 × 10^−9^	143	27

**Table 8:** Pathway enrichment analysis of Reactome signaling pathways dysregulated in MRONJ ranked according to their *p*-values. The set of seventeen hub-dysregulated genes/proteins was considered in the analysis.

Reactome Pathway ID	Name	FDR	*p*-Value	Number of Proteins in Pathway	Proteins from Gene Set
R-HSA-6785807	Interleukin-4 and interleukin-13 signaling	4.49 × 10^−8^	1.83 × 10^−10^	112	7
R-HSA-449147	Signaling by interleukins	6.39 × 10^−7^	6.95 × 10^−9^	466	9
R-HSA-6783783	Interleukin-10 signaling	6.39 × 10^−7^	7.88 × 10^−9^	47	5
R-HSA-1280215	Cytokine signaling in the immune system	1.12 × 10^−6^	1.83 × 10^−8^	730	10
R-HSA-76002	Platelet activation, signaling, and aggregation	1.47 × 10^−3^	3.37 × 10^−5^	260	5

**Table 9:** Pathway enrichment analysis of significantly dysregulated Reactome signaling pathways conducted on the set of seven miRNAs (hsa-mir-16-1, hsa-mir-21, hsa-mir-23a, hsa-mir-145, hsa-mir-186, hsa-mir-221, and hsa-mir-424) of MRONJ based on the *p*-value. Direct target genes of the set of seventeen input genes (*ALB*, *CD44*, *CXCL2*, *CXCL8*, *CXCR4*, *EEF2*, *EGF*, *IGF1*, *IL1B*, *IL6*, *ITGB1*, *JUN*, *MMP9*, *PTPRC*, *RHOA*, *TNF*, and *VEGFA*) are displayed.

Reactome Pathway ID	Name	Merged *p*-Value	Merged FDR	Term Genes	miRNAs	Direct Target Genes
R-HSA-6785807	Interleukin-4 and interleukin-13 signaling	5.9652 × 10^−33^	1.2229 × 10^−30^	122	hsa-miR-21-5p	*IL1B, VEGFA*
					hsa-miR-23a-3p	*CXCL8*
					hsa-miR-145-5p	*VEGFA*
					hsa-miR-186-5p	*VEGFA*
					hsa-miR-16-1-3p	*VEGFA*
R-HSA-449147	Signaling by interleukins	7.6257 × 10^−24^	7.8163 × 10^−22^	512	hsa-miR-21-5p	*IL1B, VEGFA*
					hsa-miR-23a-3p	*CXCL8*
					hsa-miR-145-5p	*VEGFA*
					hsa-miR-186-5p	*VEGFA*
					hsa-miR-16-1-3p	*VEGFA*
R-HSA-1643685	Diseases	2.1882 × 10^−15^	6.4083 × 10^−14^	1819	hsa-miR-21-5p	*IL1B, VEGFA*
					hsa-miR-145-5p	*VEGFA*
R-HSA-1280215	Cytokine signaling in the immune system	1.3377 × 10^−13^	2.1094 × 10^−12^	10501	hsa-miR-21-5p	*IL1B, VEGFA*
					hsa-miR-145-5p	*CD44, VEGFA*
					hsa-miR-16-1-3p	*VEGFA*
R-HSA-74160	Gene expression (transcription)	2.3944 × 10^−13^	3.2723 × 10^−12^	1661	hsa-miR-21-5p	*VEGFA*
R-HSA-9006934	Signaling by receptor tyrosine kinases	4.79563 × 10^−13^	6.14441 × 10^−12^	528	hsa-miR-21-5p	*VEGFA*
					hsa-miR-145-5p	*VEGFA*
R-HSA-212436	Generic transcription pathway	8.76149 × 10^−13^	1.05653 × 10^−11^	1372	hsa-miR-21-5p	*VEGFA*
					hsa-miR-145-5p	*VEGFA*
R-HSA-195258	RHO GTPase effectors	3.07483 × 10^−7^	1.40076 × 10^−6^	333	hsa-miR-186-3p	*ITGB1*
R-HSA-8866910	TFAP2 (AP-2) family regulates the transcription of growth factors and their receptors	2.98627 × 10^−6^	1.11306 × 10^−5^	15	hsa-miR-21-5p	*VEGFA*
					hsa-miR-145-5p	*VEGFA*
R-HSA-168256	Immune system	1.30146 × 10^−5^	3.75774 × 10^−5^	2755	hsa-miR-21-5p	*IL1B, VEGFA*
R-HSA-162582	Signal transduction	1.37359 × 10^−5^	3.91092 × 10^−5^	3138	hsa-miR-21-5p	*VEGFA*
					hsa-miR-145-5p	*VEGFA*
R-HSA-8864260	Transcriptional regulation by the AP-2 (TFAP2) family of transcription factors	2.69119 × 10^−5^	6.89618 × 10^−5^	38	hsa-miR-145-5p	*VEGFA*
R-HSA-446652	Interleukin-1 family signaling	4.46946 × 10^−4^	5.51562 × 10^−4^	165	hsa-miR-21-5p	*IL1B*
R-HSA-6783783	Interleukin-10 signaling	4.49321 × 10^−4^	5.51562 × 10^−4^	59	hsa-miR-21-5p	*IL1B*
R-HSA-1474244	Extracellular matrix organization	8.44467 × 10^−4^	9.15957 × 10^−4^	318	hsa-miR-145-5p	*CD44*
R-HSA-5660668	CLEC7A/inflammasome pathway	1.724928 × 10^−3^	1.724928 × 10^−3^	6	hsa-miR-21-5p	*IL1B*

## Data Availability

The datasets generated during and/or analyzed during the current study are available from the corresponding author upon reasonable request.
